# Supplementary material for: Human Pannexin 1 Channel is NOT Phosphorylated by Src Tyrosine Kinase at Tyr199 and Tyr309
Source: bioRxiv. 2024 Mar 18:2023.09.10.557063. Preprint. [Version 3] doi: 10.1101/2023.09.10.557063 (PMC10760007; doi:10.1101/2023.09.10.557063)

bioRxiv preprint doi: https://doi.org/10.1101/2023.09.10.557063; this version posted March 18, 2024. The copyright holder for this preprint (which was not certified by peer review) is the author/funder, who has granted bioRxiv a license to display the preprint in perpetuity. It is made available under aCC-BY 4.0 International license.

FIGURES

Figure 2—source data 1: Uncropped immunoblots for Figure 2B.
Figure 2—source data 2: Raw image for anti-PANX1-pY198 immunoblot in Figure 2B.
Figure 2—source data 3: Raw image for anti-PANX1-pY308 immunoblot in Figure 2B.
Figure 2—source data 4: Raw image for in-gel fluorescence (GFP and mCherry) in Figure 2B.

Figure 2—figure supplement 1: The hPANX1 cannot be detected by anti-PANX1-pY198 and anti-PANX1-pY308 irrespective of C-terminal GFP tag. Human PANX1 w- or w/o C-terminal GFP tag were co-expressed with mSrc-Y529F in HEK293T cells. The cell lysates were analyzed by SDS gel and blotted with anti-PANX1-pY198, anti-PANX1-pY308, anti-PANX1, and anti-Src antibodies. The in-gel fluorescence of GFP and mCherry signal were shown at the bottom. The positions of the signals detected by anti-PANX1-pY198 and anti-PANX1-pY308 antibodies did not match the position of the anti-PANX1 western blot signal and GFP fluorescence signal.

bioRxiv preprint doi: https://doi.org/10.1101/2023.09.10.557063; this version posted March 18, 2024. The copyright holder for this preprint (which was not certified by peer review) is the author/funder, who has granted bioRxiv a license to display the preprint in perpetuity. It is made available under aCC-BY 4.0 International license.

Figure 3—source data 1: Uncropped immunoblots for Figure 3.

Figure 3—source data 2: Raw image for in-gel fluorescence (GFP and mCherry) in Figure 3A.

Figure 3—source data 3: Raw image for anti-PANX1 immunoblot in Figure 3A.

Figure 3—source data 4: Raw image for anti-pY100 immunoblot in Figure 3A.

Figure 3—source data 5: Raw image for anti-Src immunoblot in Figure 3A.

Figure 3—source data 6: Raw image for anti-PANX1-pY198 immunoblot in Figure 3A.

Figure 3—source data 7: Raw image for anti-PANX1-pY308 immunoblot in Figure 3A.

Figure 3—source data 8: Raw image for in-gel fluorescence (GFP and mCherry) in Figure 3B,C.

Figure 3—source data 9: Raw image for anti-PANX1-pY308 immunoblot in Figure 3B,C.

Figure 3—source data 10: Raw image for in-gel fluorescence (GFP and mCherry) in Figure 3D.

Figure 3—source data 11: Raw image for anti-PANX1 immunoblot in Figure 3D.

Figure 3—source data 12: Raw image for anti-PANX1-pY308 immunoblot in Figure 3D.

Figure 4—source data 1: Uncropped immunoblots for Figure 4.

Figure 4—source data 2: Raw image for in-gel fluorescence (GFP and mCherry) in Figure 4A.

Figure 4—source data 3: Raw image for anti-PANX1 immunoblot in Figure 4B.

Figure 4—source data 4: Raw image for anti-Src immunoblot in Figure 4B.

Figure 4—source data 5: Raw image for in-gel fluorescence (GFP and mCherry) in Figure

bioRxiv preprint doi: https://doi.org/10.1101/2023.09.10.557063; this version posted March 18, 2024. The copyright holder for this preprint (which was not certified by peer review) is the author/funder, who has granted bioRxiv a license to display the preprint in perpetuity. It is made available under aCC-BY 4.0 International license.

4B.

Figure 4—figure supplement 1: Dephosphorylation of mSrc protein by lambda protein phosphotase (λ-PP). Phos-tag gel showed that mSrc-mCherry WT, Y529F, and K297M protein had different phosphorylation status. Phosphorylated mSrc could be converted to the non-phosphorylated form by λ-PP.

Figure 4—figure supplement 1—source data 1: Uncropped immunoblots for Figure 4—figure supplement 1.
Figure 4—figure supplement 1—source data 2: Raw image for in-gel fluorescence (mCherry) in Figure 4—figure supplement 1 (upper panel).
Figure 4—figure supplement 1—source data 3: Raw image for in-gel fluorescence (mCherry) in Figure 4—figure supplement 1 (lower panel).

Figure 4—supplement 2: Wild-type hPANX1 without C-terminal GFP tag is not phosphorylated my mSrc. Human PANX1 with/without C-terminal GFP tag is co-expressed with Src-mCherry WT, Y529F, or K297M in HEK293T cells. Cell lysate was subjected to PNGase F de-glycosylation prior to analysis on SDS-PAGE gel (upper and middle panels) or Phos-tag gel (bottom panel). Upper panel showed the GFP and mCherry fluorescence signal in SDS-PAGE gel. Middle panel showed the western blot result of anti-PANX1 antibody blot in SDS-PAGE gel. Bottom panel showed the western blot result of anti-PANX1 antibody blot in Phos-tag gel.

Figure 4—figure supplement 2—source data 1: Uncropped immunoblots for Figure 4—figure supplement 2.
Figure 4—figure supplement 2—source data 2: Raw image for in-gel fluorescence (GFP and mCherry) in Figure 4—figure supplement 2 (upper panel).
Figure 4—figure supplement 2—source data 3: Raw image for anti-PANX1 immunoblot in Figure 4—figure supplement 2 (middle panel).
Figure 4—figure supplement 2—source data 4: Raw image for anti-PANX1 immunoblot in Figure 4—figure supplement 2 (lower panel).

Figure 4—supplement 3: Coverage map of LC-MS/MS analysis of purified hPANX1-GFP. A, The sequence coverage of hPANX1 expressed alone. **B**, The sequence coverage of hPANX1 expressed with the constitutively active mSrc Y529F. Of note, both Tyr199 and Tyr309 are covered by LC-MS/MS spectra.

Figure 4—figure supplement 3—source data 1: LC-MS/MS data for WT human PANX1 with or without co-expressing Src-Y529F mutant [Dataset]. Dryad. https://doi.org/10.5061/dryad.4tmpg4fh7

Figure 4—supplement 4: Representative LC-MS/MS spectra containing the Tyr199 of hPANX1. A, The raw spectrum of (K)YPIVEQYLKTK(K) peptide from hPANX1 expressed without Src. B ions and Y ions are colored in red and blue, respectively. **B**, The fragmentation table of the (K)YPIVEQYLKTK(K) peptide shown in panel A. Red and blue color highlighted the

bioRxiv preprint doi: https://doi.org/10.1101/2023.09.10.557063; this version posted March 18, 2024. The copyright holder for this preprint (which was not certified by peer review) is the author/funder, who has granted bioRxiv a license to display the preprint in perpetuity. It is made available under aCC-BY 4.0 International license.

B ions and Y ions for the detected amino acid, respectively. The non-phosphorylated Tyr199 is supported by both B ions and Y ions (blue frame). **C**, The raw spectrum of (K)YPIVEQYLK(T) peptide from human PANX1 expressed with mSrc Y529F mutant. B ions and Y ions are colored in red and blue, respectively. **D**, The fragmentation table of the the (K)YPIVEQYLK(T) peptide shown in panel C. Red and blue color highlighted the B ions and Y ions for the detected amino acid. The non-phosphorylated Tyr199 is supported by Y ions (blue frame).

Figure 4—supplement 5: Representative LC-MS/MS spectrum covering the Tyr309 for hPANX1. A, The raw spectrum of (K)VYEILPTFDVLHFK(S) peptide from hPANX1 expressed without Src. B ions and Y ions are colored in red and blue, respectively. **B**, The fragmentation table of the (K)VYEILPTFDVLHFK(S) peptide shown in A. Red and blue color highlighted the B ions and Y ions for the detected amino acid, respectively. The non-phosphorylated Tyr308 is supported B ions (blue frame). **C**, The raw spectrum of (K)VYEILPTFDVLHFK(S) peptide from human PANX1 expressed with mSrcY529F mutant. B ions and Y ions are colored in red and blue, respectively. **D**, The fragmentation table of the the (K)VYEILPTFDVLHFK(S) peptide shown in C. Red and blue color highlighted the B ions and Y ions for the detected amino acid. The non-phosphorylated Tyr309 is supported by both B ions and Y ions (blue frame).

Figure 4—supplement 6: Representative LC-MS/MS spectrum covering the Ser385 for hPANX1. A, The raw spectrum of (K)TPMSAEMR(E) peptide from hPANX1 expressed without Src. B ions and Y ions are colored in red and blue, respectively. **B**, The fragmentation table of the (K)TPMSAEMR(E) peptide shown in A. Red and blue color highlighted the B ions and Y ions for the detected amino acid, respectively. The non-phosphorylated Ser385 is supported by both B ions and Y ions (blue frame). **C**, One raw spectrum of (K)TPMSAEMR(E) peptide from human PANX1 expressed with Src-Y529F mutant. B ions and Y ions are colored in red and blue, respectively. **D**, The fragmentation table of the (K)TPMSAEMR(E) peptide shown in C. Red and blue color highlighted the B ions and Y ions for the detected amino acid. The phosphorylated Ser385 is supported by Y ions (blue frame). **E**, One raw spectrum of (K)TPMSAEMR(E) peptide from hPANX1 expressed with Src-Y529F mutant. B ions and Y ions are colored in red and blue, respectively. **F**, The fragmentation table of the (K)TPMSAEMR(E) peptide shown in E. Red and blue color highlighted the B ions and Y ions for the detected amino acid. The non-phosphorylated Ser385 is supported by both B ions and Y ions (blue frame).

Figure 5—source data 1: Uncropped immunoblots for Figure 5.
Figure 5—source data 2: Raw image for in-gel fluorescence (GFP and mCherry) in Figure 5A.
Figure 5—source data 3: Raw image for in-gel fluorescence (GFP and mCherry) in Figure 5B.

bioRxiv preprint doi: https://doi.org/10.1101/2023.09.10.557063; this version posted March 18, 2024. The copyright holder for this preprint (which was not certified by peer review) is the author/funder, who has granted bioRxiv a license to display the preprint in perpetuity. It is made available under aCC-BY 4.0 International license.

Figure 6—source data 1: Uncropped immunoblots for Figure 6.
Figure 6—source data 2: Raw image for in-gel fluorescence (GFP and mCherry) in Figure 6A.
Figure 6—source data 3: Raw image for anti-PANX1 immunoblot in Figure 6B.
Figure 6—source data 4: Raw image for anti-Src immunoblot in Figure 6B.
Figure 6—source data 5: Raw image for in-gel fluorescence (GFP and mCherry) in Figure 6B.

Figure 6—supplement 1: The anti-PANX1 antibody detects both hPANX1 and non-specific proteins. The non-transfected HEK293T cells or cells transfected with PANX1 (without the GFP tag) is digested by PNGase F and analyzed by western blot. The anti-PANX1 antibody produced two bands from the non-transfected HEK293T cell lysate at approximately 100 kDa and 50 kDa. After PNGase F treatment, the 50 kDa band (indicated by blue dotted lines) can be partially shifted to a location where monomeric PANX1 protein is located. The 100 kDa band (indicated by dotted magenta lines) is not sensitive to PNGase F treatment. The right panel showed a long exposure image of the same blot on the left.

Figure 6—figure supplement 1—source data 1: Uncropped immunoblots for Figure 6—figure supplement 1.
Figure 6—figure supplement 1—source data 2: Raw image for anti-PANX1 immunoblot in Figure 6—figure supplement 1 (left panel).
Figure 6—figure supplement 1—source data 3: Raw image for anti-PANX1 immunoblot in Figure 6—figure supplement 1 (right panel).

bioRxiv preprint doi: https://doi.org/10.1101/2023.09.10.557063; this version posted March 18, 2024. The copyright holder for this preprint (which was not certified by peer review) is the author/funder, who has granted bioRxiv a license to display the preprint in perpetuity. It is made available under aCC-BY 4.0 International license.

Figure 2-figure supplement 1

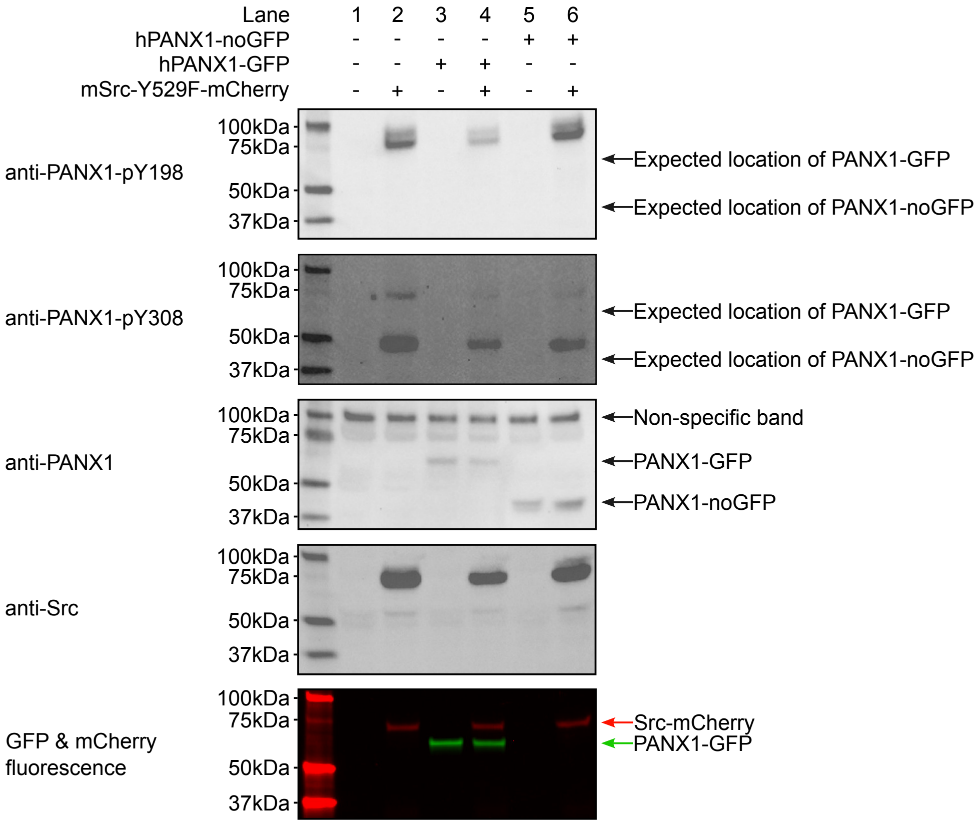

bioRxiv preprint doi: https://doi.org/10.1101/2023.09.10.557063; this version posted March 18, 2024. The copyright holder for this preprint (which was not certified by peer review) is the author/funder, who has granted bioRxiv a license to display the preprint in perpetuity. It is made available under aCC-BY 4.0 International license.

Figure 4-figure supplement 1

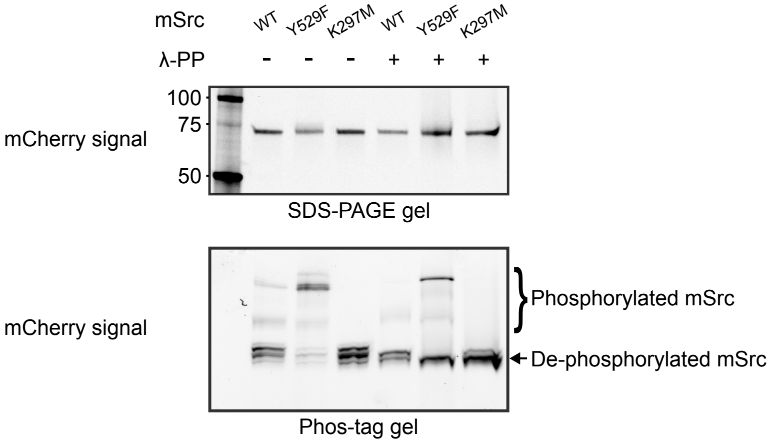

bioRxiv preprint doi: https://doi.org/10.1101/2023.09.10.557063; this version posted March 18, 2024. The copyright holder for this preprint (which was not certified by peer review) is the author/funder, who has granted bioRxiv a license to display the preprint in perpetuity. It is made available under aCC-BY 4.0 International license.

Figure 4-figure supplement 2

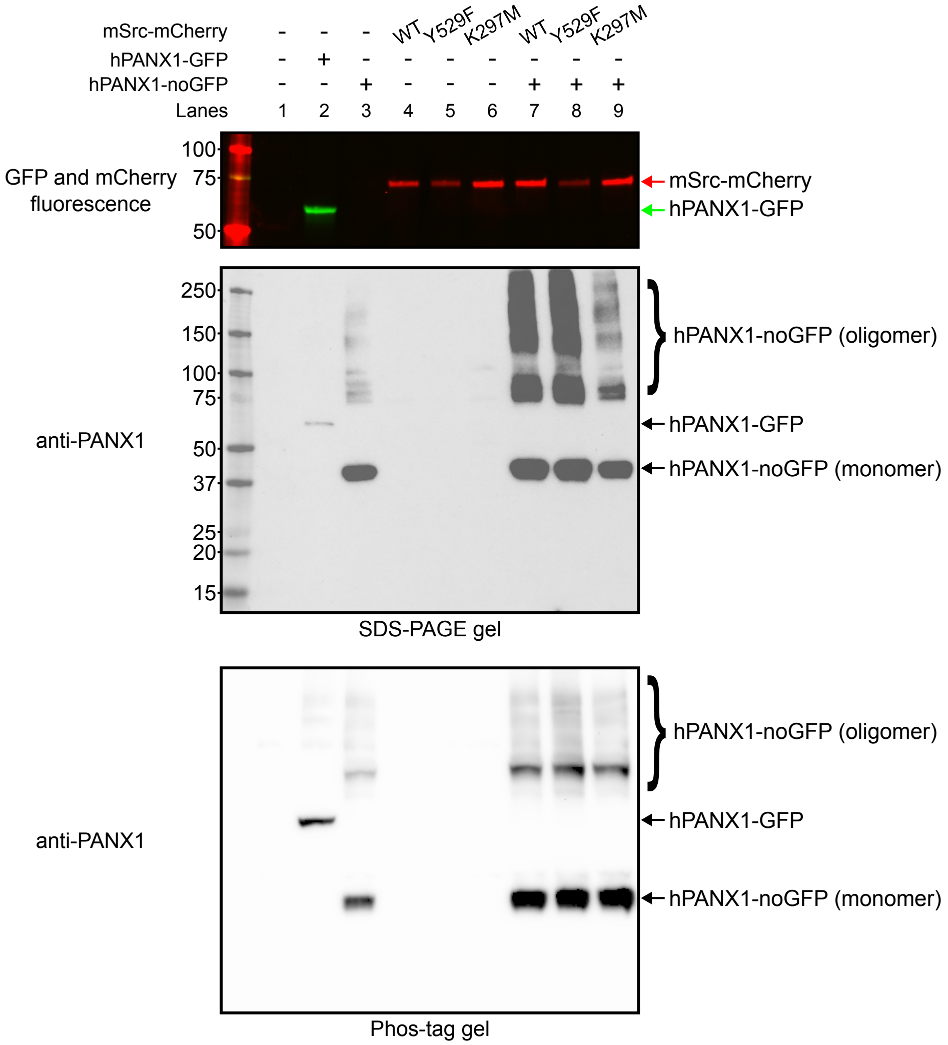

bioRxiv preprint doi: https://doi.org/10.1101/2023.09.10.557063; this version posted March 18, 2024. The copyright holder for this preprint (which was not certified by peer review) is the author/funder, who has granted bioRxiv a license to display the preprint in perpetuity. It is made available under aCC-BY 4.0 International license.

Figure 4-figure supplement 3

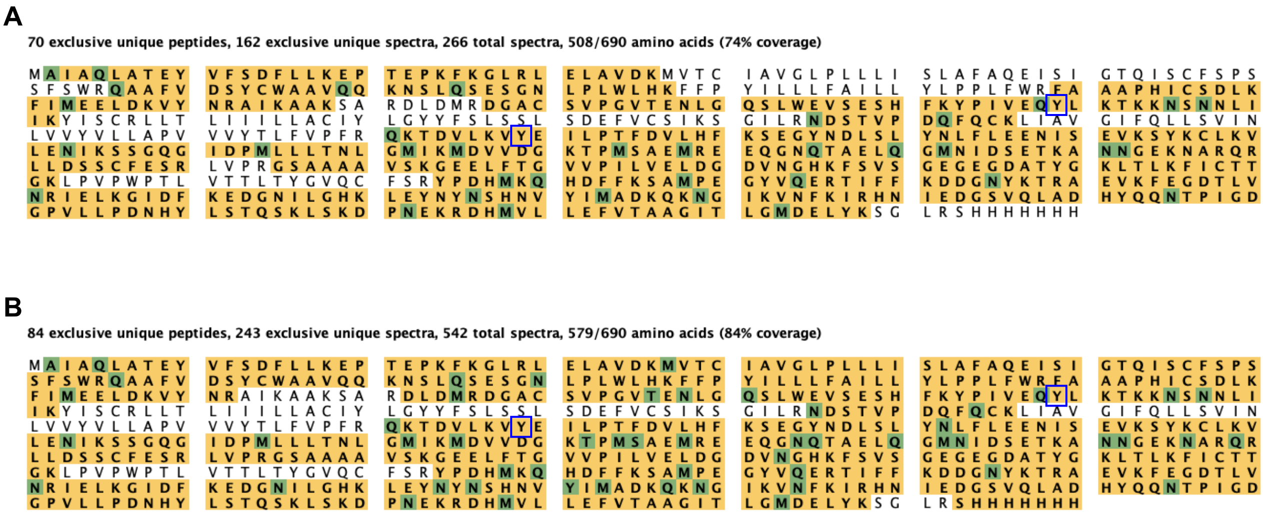

bioRxiv preprint doi: https://doi.org/10.1101/2023.09.10.557063; this version posted March 18, 2024. The copyright holder for this preprint (which was not certified by peer review) is the author/funder, who has granted bioRxiv a license to display the preprint in perpetuity. It is made available under aCC-BY 4.0 International license.

Figure 4-figure supplement 4

A

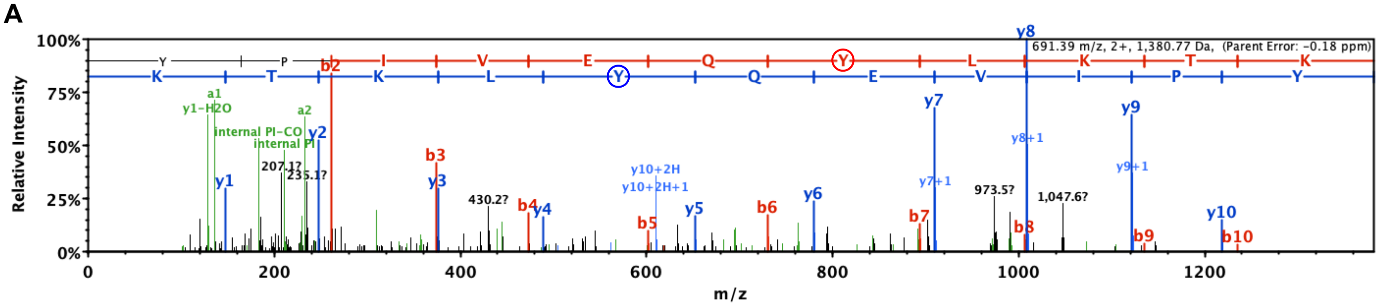

B

B	B Ions	B+2H	B-NH3	B-H2O	AA	Y Ions	Y+2H	Y-NH3	Y-H2O	Y
1	164.1			146.1	Y	1,381.8	691.4	1,364.7	1,363.8	11
2	261.1			243.1	P	1,218.7	609.9	1,201.7	1,200.7	10
3	374.2			356.2	I	1,121.7	561.3	1,104.6	1,103.6	9
4	473.3			455.3	V	1,008.6	504.8	991.5	990.6	8
5	602.3			584.3	E	909.5	455.3	892.5	891.5	7
6	730.4	365.7	713.4	712.4	Q	780.5	390.7	763.4	762.5	6
7	893.4	447.2	876.4	875.4	Y	652.4	326.7	635.4	634.4	5
8	1,006.5	503.8	989.5	988.5	L	489.3	245.2	472.3	471.3	4
9	1,134.6	567.8	1,117.6	1,116.6	K	376.3	188.6	359.2	358.2	3
10	1,235.7	618.3	1,218.6	1,217.7	T	248.2		231.1	230.1	2
11	1,381.8	691.4	1,364.7	1,363.8	K	147.1		130.1	129.1	1

C

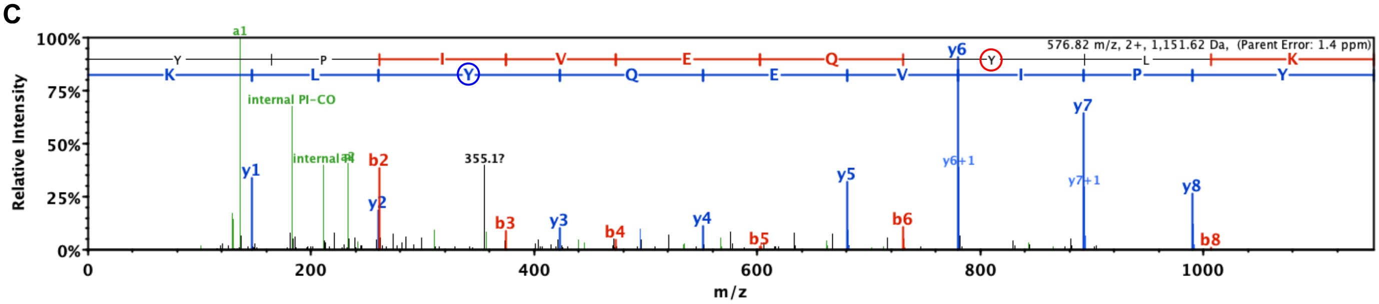

D

B	B Ions	B+2H	B-NH3	B-H2O	AA	Y Ions	Y+2H	Y-NH3	Y-H2O	Y
1	164.1			146.1	Y	1,152.6	576.8	1,135.6	1,134.6	9
2	261.1			243.1	P	989.6	495.3	972.5	971.6	8
3	374.2			356.2	I	892.5	446.8	875.5	874.5	7
4	473.3			455.3	V	779.4	390.2	762.4	761.4	6
5	602.3			584.3	E	680.4		663.3	662.4	5
6	730.4	365.7	713.4	712.4	Q	551.3		534.3	533.3	4
7	893.4	447.2	876.4	875.4	Y	423.3		406.2	405.2	3
8	1,006.5	503.8	989.5	988.5	L	260.2		243.2	242.2	2
9	1,152.6	576.8	1,135.6	1,134.6	K	147.1		130.1	129.1	1

bioRxiv preprint doi: https://doi.org/10.1101/2023.09.10.557063; this version posted March 18, 2024. The copyright holder for this preprint (which was not certified by peer review) is the author/funder, who has granted bioRxiv a license to display the preprint in perpetuity. It is made available under aCC-BY 4.0 International license.

Figure 4-figure supplement 5

A

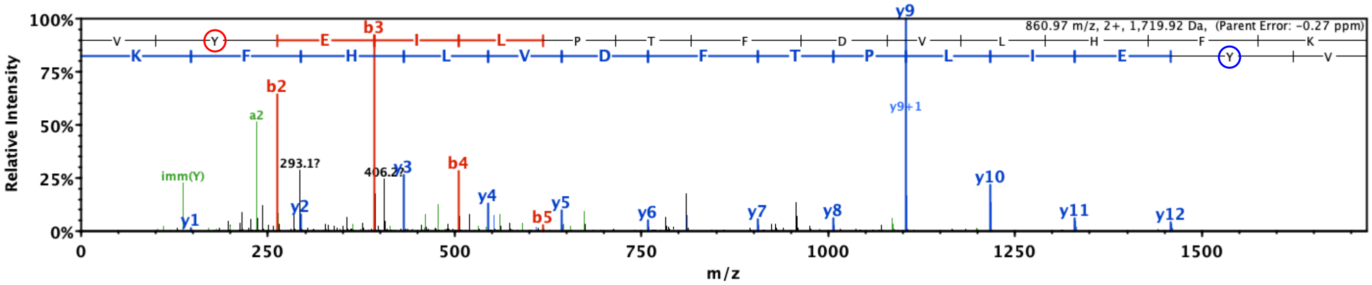

B

B	B Ions	B+2H	B-NH3	B-H2O	AA	Y Ions	Y+2H	Y-NH3	Y-H2O	Y
1	100.1				V	1,720.9	861.0	1,703.9	1,702.9	14
2	263.1			245.1	Y	1,621.9	811.4	1,604.8	1,603.9	13
3	392.2			374.2	E	1,458.8	729.9	1,441.8	1,440.8	12
4	505.3			487.3	I	1,329.8	665.4	1,312.7	1,311.7	11
5	618.3			600.3	L	1,216.7	608.8	1,199.6	1,198.7	10
6	715.4	358.2		697.4	P	1,103.6	552.3	1,086.6	1,085.6	9
7	816.5	408.7		798.4	T	1,006.5	503.8	989.5	988.5	8
8	963.5	482.3		945.5	F	905.5	453.2	888.5	887.5	7
9	1,078.5	539.8		1,060.5	D	758.4	379.7	741.4	740.4	6
10	1,177.6	589.3		1,159.6	V	643.4	322.2	626.4	625.4	5
11	1,290.7	645.9		1,272.7	L	544.3	272.7	527.3	526.3	4
12	1,427.8	714.4		1,409.7	H	431.2	216.1	414.2	413.2	3
13	1,574.8	787.9		1,556.8	F	294.2		277.2	276.2	2
14	1,720.9	861.0	1,703.9	1,702.9	K	147.1		130.1		1

C

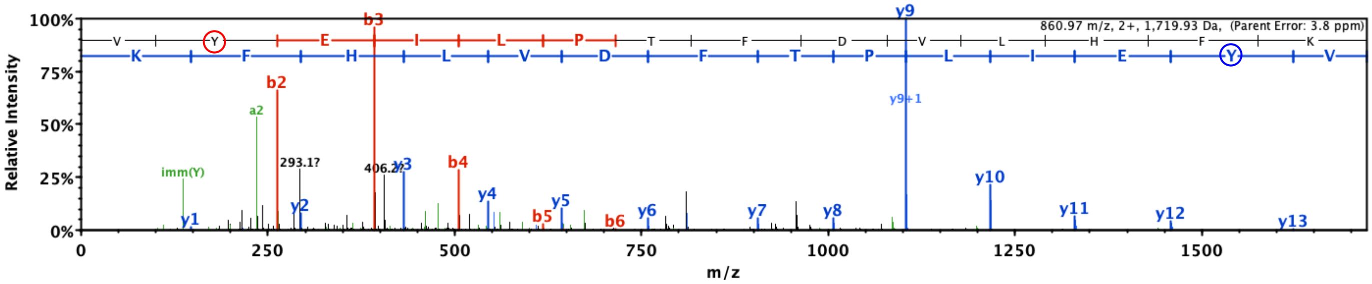

D

B	B Ions	B+2H	B-NH3	B-H2O	AA	Y Ions	Y+2H	Y-NH3	Y-H2O	Y
1	100.1				V	1,720.9	861.0	1,703.9	1,702.9	14
2	263.1			245.1	Y	1,621.9	811.4	1,604.8	1,603.9	13
3	392.2			374.2	E	1,458.8	729.9	1,441.8	1,440.8	12
4	505.3			487.3	I	1,329.8	665.4	1,312.7	1,311.7	11
5	618.3			600.3	L	1,216.7	608.8	1,199.6	1,198.7	10
6	715.4	358.2		697.4	P	1,103.6	552.3	1,086.6	1,085.6	9
7	816.5	408.7		798.4	T	1,006.5	503.8	989.5	988.5	8
8	963.5	482.3		945.5	F	905.5	453.2	888.5	887.5	7
9	1,078.5	539.8		1,060.5	D	758.4	379.7	741.4	740.4	6
10	1,177.6	589.3		1,159.6	V	643.4	322.2	626.4	625.4	5
11	1,290.7	645.9		1,272.7	L	544.3	272.7	527.3	526.3	4
12	1,427.8	714.4		1,409.7	H	431.2	216.1	414.2	413.2	3
13	1,574.8	787.9		1,556.8	F	294.2		277.2	276.2	2
14	1,720.9	861.0	1,703.9	1,702.9	K	147.1		130.1		1

bioRxiv preprint doi: https://doi.org/10.1101/2023.09.10.557063; this version posted March 18, 2024. The copyright holder for this preprint (which was not certified by peer review) is the author/funder, who has granted bioRxiv a license to display the preprint in perpetuity. It is made available under aCC-BY 4.0 International license.

Figure 4-figure supplement 6

A

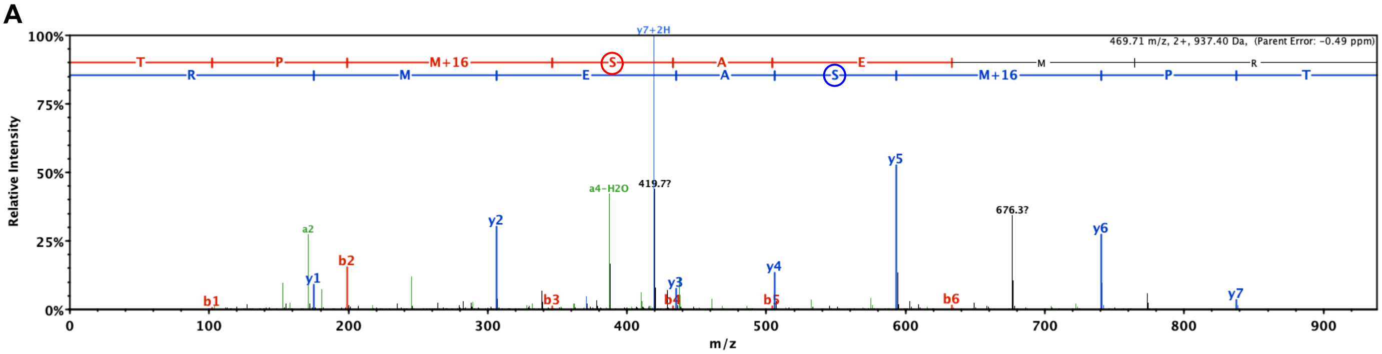

B

B	B Ions	B+2H	B-NH3	B-H2O	AA	Y Ions	Y+2H	Y-NH3	Y-H2O	Y
1	102.1			84.0	T	938.4	469.7	921.4	920.4	8
2	199.1			181.1	P	837.4	419.2	820.3	819.3	7
3	346.1			328.1	M+16	740.3	370.7	723.3	722.3	6
4	433.2			415.2	S	593.3		576.2	575.3	5
5	504.2			486.2	A	506.2		489.2	488.2	4
6	633.3	317.1		615.2	E	435.2		418.2	417.2	3
7	764.3	382.7		746.3	M	306.2		289.1		2
8	938.4	469.7	921.4	920.4	R	175.1		158.1		1

C

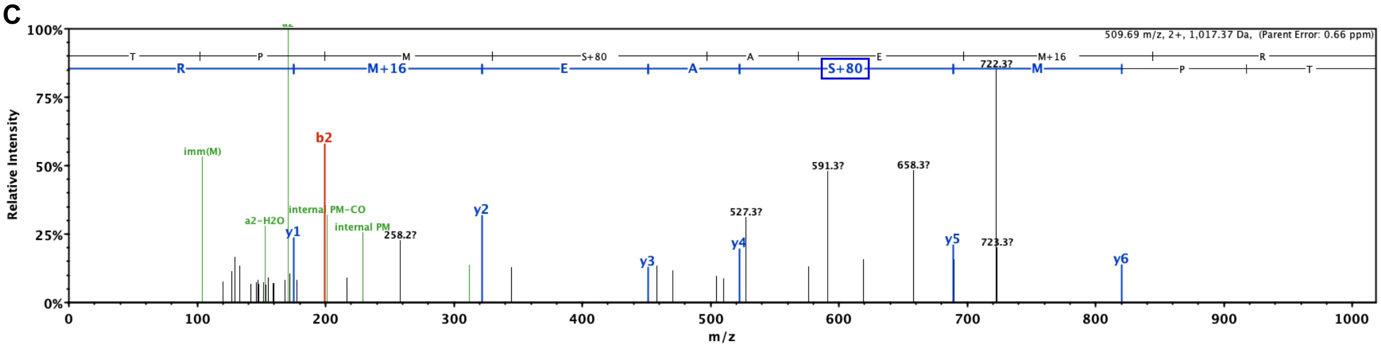

D

B	B Ions	B+2H	B-NH3	B-H2O	AA	Y Ions	Y+2H	Y-NH3	Y-H2O	Y
1	102.1			84.0	T	1,018.4	509.7	1,001.3	1,000.4	8
2	199.1			181.1	P	917.3	459.2	900.3	899.3	7
3	330.1			312.1	M	820.3	410.6	803.2	802.3	6
4	497.1			479.1	S+80	689.2		672.2	671.2	5
5	568.2			550.2	A	522.2		505.2	504.2	4
6	697.2	349.1		679.2	E	451.2		434.2	433.2	3
7	844.3	422.6		826.3	M+16	322.2		305.1		2
8	1,018.4	509.7	1,001.3	1,000.4	R	175.1		158.1		1

E

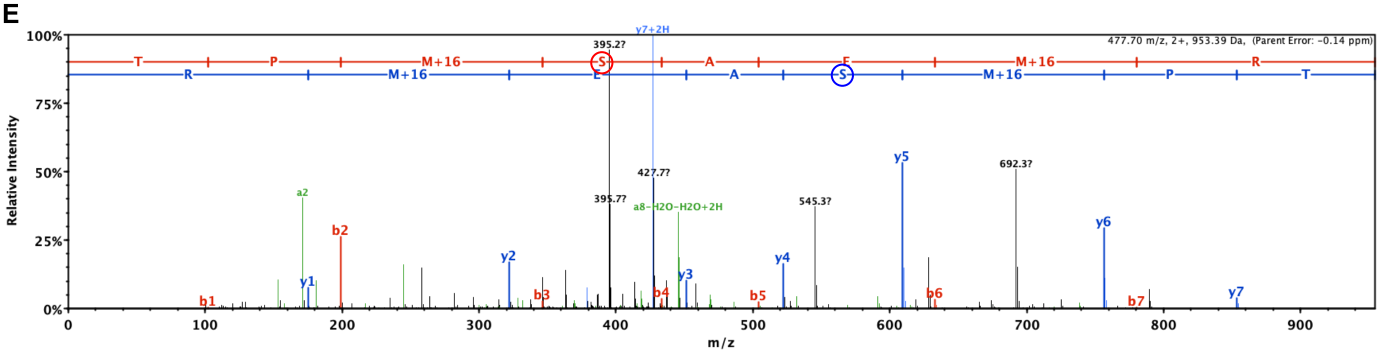

F

B	B Ions	B+2H	B-NH3	B-H2O	AA	Y Ions	Y+2H	Y-NH3	Y-H2O	Y
1	102.1			84.0	T	954.4	477.7	937.4	936.4	8
2	199.1			181.1	P	853.4	427.2	836.3	835.3	7
3	346.1			328.1	M+16	756.3	378.7	739.3	738.3	6
4	433.2			415.2	S	609.3		592.2	591.3	5
5	504.2			486.2	A	522.2		505.2	504.2	4
6	633.3	317.1		615.2	E	451.2		434.2	433.2	3
7	780.3	390.6		762.3	M+16	322.2		305.1		2
8	954.4	477.7	937.4	936.4	R	175.1		158.1		1

bioRxiv preprint doi: https://doi.org/10.1101/2023.09.10.557063; this version posted March 18, 2024. The copyright holder for this preprint (which was not certified by peer review) is the author/funder, who has granted bioRxiv a license to display the preprint in perpetuity. It is made available under aCC-BY 4.0 International license.

Figure 6-figure supplement 1

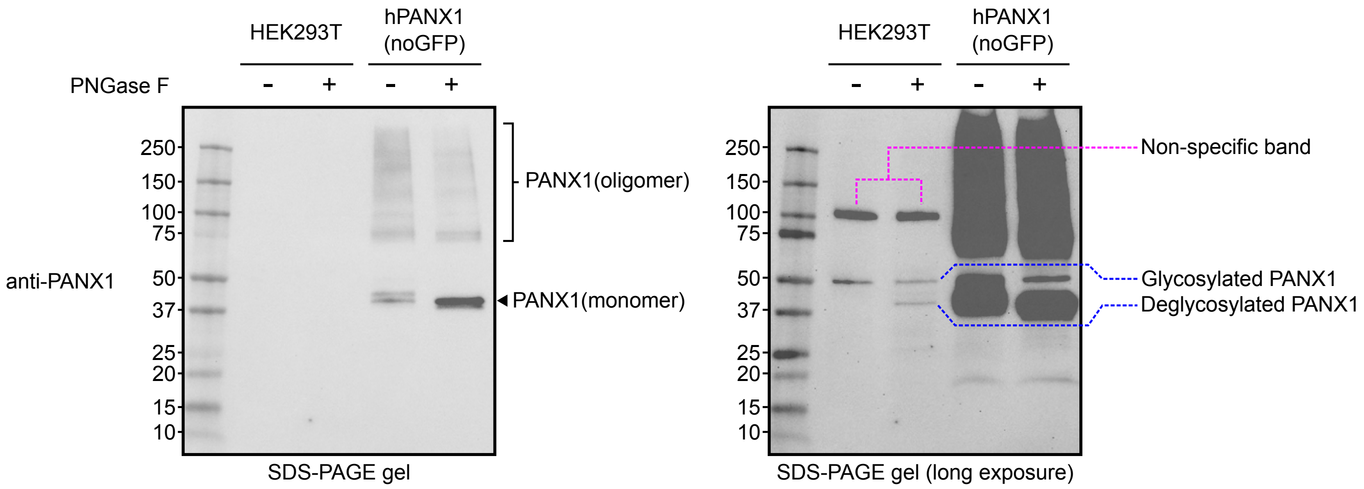

Supplement: Supplement 1 [file NIHPP2023.09.10.557063v3-supplement-1.pdf]
